# 

**Published:** 1949-10

**Authors:** 


### CONTENTS

| | Page |
|---|---|
| **Unusual Children.** I.—Congenital Syphilis with Gangrene of the Extremities. II.—Dwarfism. III.—Progeria: By A. V. Neale, M.D., F.R.C.P. | 89 |
| IV.—Cretinism. V.—Mongolism: By Beryl D. Corner, M.D., M.R.C.P. | 91 |
| VI.—Gargoylism: By John Lowe, M.D. F.R.C.S. | 92 |
| **The Prophylactic Uses of Chemotherapy.** By J. A. Boycott, D.M. | 95 |
| **Thymectomy for Myasthenia Gravis.** By Geoffrey Keynes M.D., F.R.C.S. | 100 |
| **Editorial Notes** | 103 |
| **Obituaries** | 104 |
| **Reviews of Books** | 106 |
| **Meetings of Societies** | 109 |
| **Local Medical Notes** | 111 |

